# Supervised exercise-based rehabilitation for people with intermittent claudication–Study protocol for a Danish implementation process (StRiDE)

**DOI:** 10.1371/journal.pone.0315577

**Published:** 2025-01-13

**Authors:** Sara Fredslund Hajdú, Helle Bøgard, Thomas Vedste Aagaard, Stine Gundtoft Roikjær, Charlotte Simonÿ, Anne Dalhoff, Kim Houlind, Alexander Luijk, Ida Ulriksen, Lotte Therkildsen Jensen, Søren T. Skou, Lars Hermann Tang

**Affiliations:** 1 The Research and Implementation Unit PROgrez, Department of Physiotherapy and Occupational Therapy Næstved-Slagelse-Ringsted Hospitals, Slagelse, Denmark; 2 The Department of Regional Health Research, University of Southern Denmark, Odense, Denmark; 3 Center for Neurological Research, Næstved-Slagelse-Ringsted Hospitals, Slagelse, Denmark; 4 Department of Vascular Surgery, Lillebaelt Hospital, Kolding and Department of Regional Health Research, University of Southern Denmark, Odense, Denmark; 5 Department of Sports Science and Clinical Biomechanics, Center for Muscle and Joint Health, University of Southern Denmark, Odense, Denmark; Yale University School of Medicine, UNITED STATES OF AMERICA

## Abstract

**Introduction:**

Intermittent claudication is a peripheral artery disease caused by arteriosclerosis. People with intermittent claudication experience leg cramping during walking, with relief of symptoms during rest. Evidence shows that by participating in supervised exercise therapy and smoking cessation programs, people with intermittent claudication can reduce those symptoms and improve health-related quality of life and maximal walking distance while minimizing the need for an operation. However, implementation of such health-promoting initiatives in clinical practice in Denmark and other countries is limited. This is a protocol presenting the implementation process of supervised exercise therapy and smoking cessation in a region of Denmark.

**Methods and analysis:**

The implementation process is a collaboration between the municipalities in the Region of Zealand and the Department of Vascular Surgery at University Hospital Zealand. The study uses a convergent mixed-methods prospective clinical cohort design, and the theoretical frame of this implementation process follows the framework for Adapting an existing intervention to a new context (ADAPT). The process involves stakeholder engagement, ongoing evaluation through key performance indicators and relevant outcomes that will inform the implementation process across and within each municipality.

**Dissemination:**

Dissemination will happen throughout the process through continued meetings with stakeholders and dissemination of performance indicators and outcome results obtained through a database. All information about the study and material will be freely available. The project is registred on Clinicalgov (NCT06299956).

## Introduction

### Background and rationale

More than 200 million people worldwide have peripheral arterial disease (PAD) [1]. PAD is an arterial occlusive disease caused by arteriosclerosis. The consequences for people with PAD may be a progression from asymptomatic disease to intermittent claudication (IC), critical limb ischemia and finally amputation [2]. Progression of the disease entails a negative impact on health-related quality of life (HRQoL) and can eventually lead to death [2]. Patients with IC have a five-year all-cause mortality rate of 10–15% and a 20% chance of a non-fatal cardiovascular event [3]. When IC progresses to critical limb ischemia, an even higher mortality rate of 25% after one year has been reported [4].

The common treatment regime for IC is walking advice, medical treatment and surgical intervention, mainly endovascular revascularization for progressive PAD [2]. However, early revascularization is associated with a greater risk of subsequent requirement for major amputation than initial conservative treatment [5].

The European guidelines recommend supervised exercise therapy (SET), smoking cessation and a healthy diet for individuals with PAD [6]. These components can increase walking distance, reduce leg pain, minimize the need for an operation and improve HRQoL in people with IC [2, 7–9]. Only when daily activities are severely compromised should revascularization be considered [6].

Despite the evident impact of SET on IC, [2] implementation remains scarce in most European countries [10]. A survey from 2019 showed that 96% of vascular surgeons in Denmark would refer their patients to SET if it was accessible [11]. Despite this, only 14% of the 98 municipalities in Denmark provide systematic SET targeting IC [11] leaving the majority of Danish patients with simple advice on ways to increase physical activity by themselves, with no professional supervision [11]. A cost-benefit analysis of the implementation of a stepped care model consisting of primary SET treatment followed by revascularization in case of SET failure found significant potential annual savings in Dutch healthcare resources between 3.9–33.0 million euros [9]. Altogether, this highlights the potential impact on patients and society of implementing SET for people with IC.

Therefore, in 2022, the Regional Council of Zealand allocated funds to implement SET as part of routine practice for people with IC. Region Zealand has a total population of 836,000 people, with approximately 10,000 of these living with IC and an estimated 1500 new cases of IC each year [12]. The purpose of the implementation is to ensure that all citizens with IC in Region Zealand are referred to a rehabilitation intervention including, as a minimum, SET and smoking cessation. This article describes and details the protocol for implementing SET and smoking cessation in Region Zealand in Denmark.

### Objective

The objective of this paper is to describe:

1. The development and design of the implementation process of a rehabilitative intervention including SET and smoking cessation in Region Zealand with a 6-month follow-up period after completion of the program.
2. The ongoing quality monitoring process of the implementation process through key performance indicators addressing referral, recruitment, retention, data completeness, intervention delivery and attendance and to collect feedback that will guide refinements of the intervention delivery and data collection.
3. Outcomes available for assessment of benefits and harms from the SET intervention.

## Methods

### Study design and theoretical approach

The study uses a convergent mixed-methods prospective clinical cohort approach [13].

**Fig 1:**
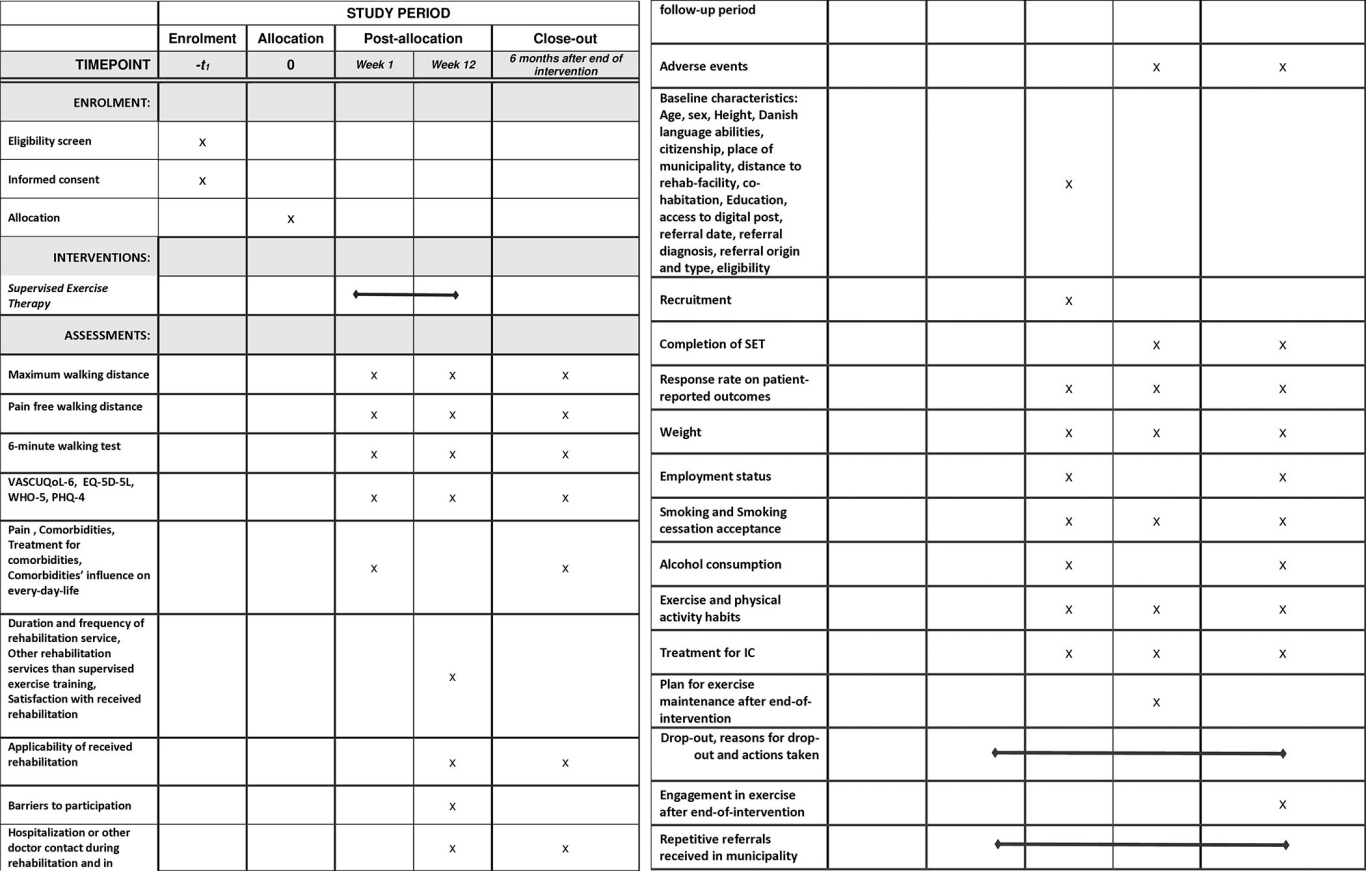
The SPIRIT figure of enrolment, interventions and assessments.

The design and theoretical foundation of the implementation process are guided by the framework for Adapting an existing intervention to a new context (ADAPT) [14]. The ADAPT framework focuses specifically on the systematic course and actions needed to adapt and/or transfer an existing intervention to a new context. It also focuses on involving stakeholders, the process of planning and undertaking adaptations. Finally, it focuses on evaluating and implementing adapted interventions in routine practice and reporting adaption processes and outcomes to secure the transfer and adaptation of the intervention [14]. This protocol is written in consensus with the SPIRIT statement that defines standard protocol items for clinical trials [15]. Fig 1 is the SPIRIT schedule of enrolment, interventions and assessments.

#### Setting of implementations

**Fig 2:**
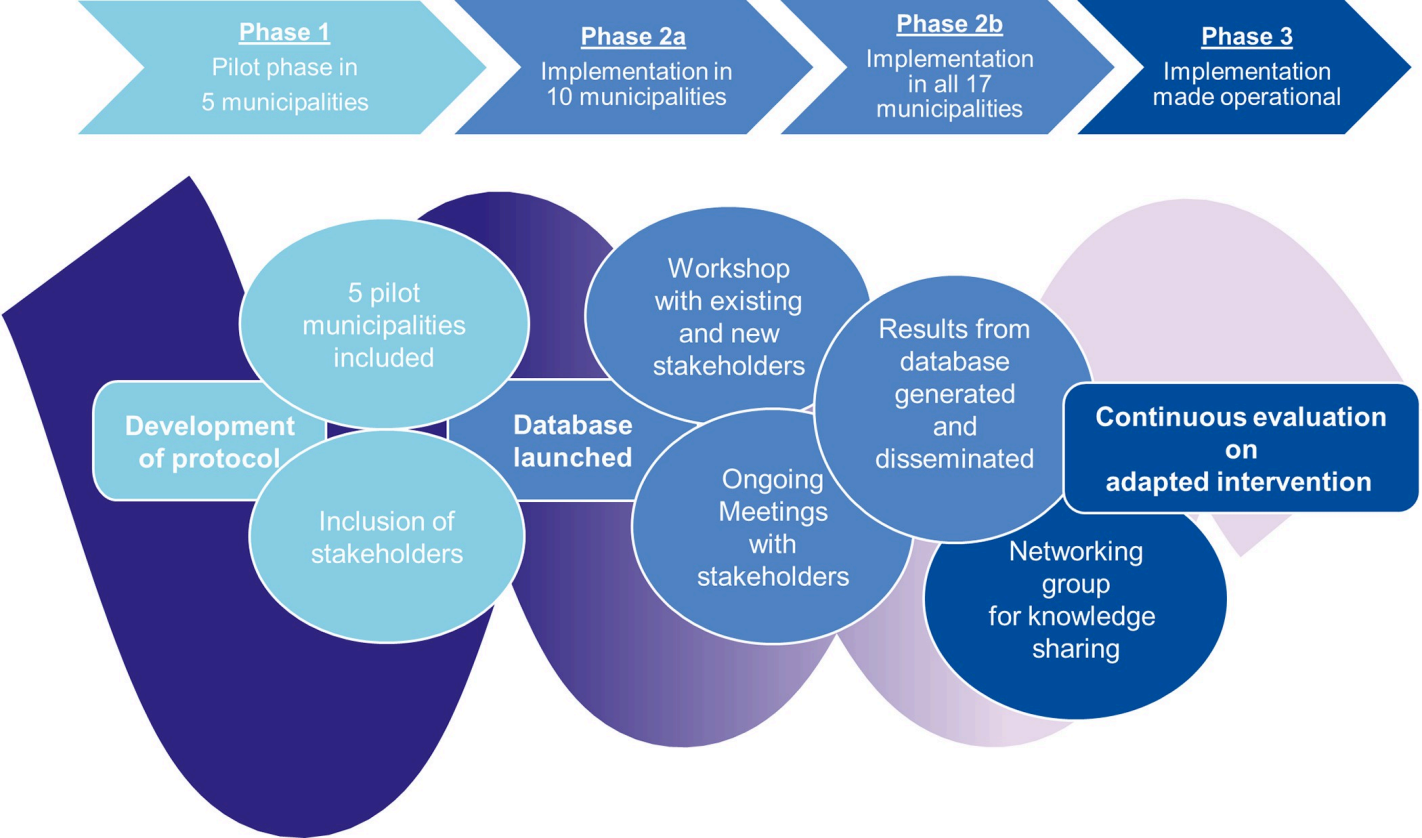
The implementation process.

The SET intervention and smoking cessation will take place in a municipal setting, geographically close to where people with IC live. The period for recruitment is 1 Sept. 2023–31 Dec. 2025. The intervention was inspired by the studies of Hageman et al., Murphy et al. 2008 and Murphy et al. 2012 [2, 7, 16]. Denmark is divided into five regions, each divided into a number of municipalities with a different number of local healthcare centers. These centers hold a main responsibility for rehabilitation services in Denmark. The 17 municipalities in the Region of Zealand are organized into four clusters that facilitate political and strategic intersectoral collaboration. To accommodate this organizational structure and ensure a geographical distribution, a minimum of one municipality in each cluster will be included in the pilot phase. The implementation process outlined in this protocol consists of three phases, as depicted in Fig 2. The first phase, known as the pilot phase, includes five municipalities and is expected to take place in 2023 and 2024. The second phase expected to take place in 2024, focuses on including the rest of the municipalities, where some municipalities have more than one rehabilitation site. Finally, in the third phase, the implementation of SET becomes a part of routine practice in the municipalities and the focus is on maintaining the adapted intervention. This phase is thought to run throughout 2025 and forwards.

#### Patient and public involvement

Engaging patients in the research process has the potential to better capture the patients’ perspectives [17] and is relevant to ensure that the study obtains a high level of relevance to patients and the public [18, 19]. The Patient Association for Amputees in Denmark [20] was involved in the design of this study and offered insights on barriers and facilitators. The research questions and outcomes measured were developed by a combination of research literature and involvement from all stakeholders e.g. surgeons, representatives of the municipalities as well as patients. To evaluate the burden of the questionnaires and tests in the SET, the participants will be asked in an interview how burdensome completion of the questionnaires was. All stakeholders including participants will be involved in the plan for dissemination of the results.

#### Stakeholder involvement

In all phases of this implementation process stakeholder involvement is a key factor as underlined by the ADAPT guideline [14] and this process is theoretically guided by Eskerod and methodically by Bitner, Ostrom & Morgan [21, 22]. The implementation process will unfold in close collaboration with the municipalities and the Department of Vascular Surgery at University Hospital Zealand (SUH). Furthermore, clinicians from the Southern Region of Denmark with experience in implementing SET for patients with IC will be sparring partners [23]. A networking group will be formed, including vascular surgeons, managers, physiotherapists, and smoking cessation consultants from the five pilot municipalities. Regular status and consensus meetings are planned to ensure the exchange of the experiences made and ongoing dialogue on the development and adjustments of the intervention, including discussions on how to merge research evidence and real-life context in the municipalities. Physiotherapists delivering the program will attend at least one on-site session addressing how to conduct the clinical tests and perform SET as well as on-site supervision. If deemed necessary by the project team and the physiotherapists delivering the SET, further in-person or online meetings will be scheduled.

#### Eligibility

The target group of the intervention is citizens of Region Zealand diagnosed with IC. Patients are recruited from the Department of Vascular Surgery at SUH and referred to municipality-based rehabilitation via a regular electronic rehabilitation plan, cf. the Danish Health Act §140 [24]. Eligibility for participating in SET is always evaluated by a vascular surgeon, and patients with contraindications e.g. medical issues or comorbidity that precludes participation in SET are not referred. Participants can be newly diagnosed patients or patients who have already undergone vascular surgery. Patients are referred by their general practitioner to the Department of Vascular Surgery based on potential ischemic indications. Criteria for referral were articulated in close collaboration with referring vascular surgeons as follows:

### Criteria for referral

- Functional impairments and disabilities due to intermittent claudication. Often related to leg pain when walking and relief of symptoms during rest.
- Distal blood pressure with an ankle-brachial index (ABI) > 50 mmHg for non-diabetics or toe brachial index (TBI) > 40 mmHg for diabetics.

As part of the implementation process, we are investigating other pathways for referrals in this Danish setting, e.g. directly from the general practitioner.

#### Recruitment and participant timeline

**Fig 3:**
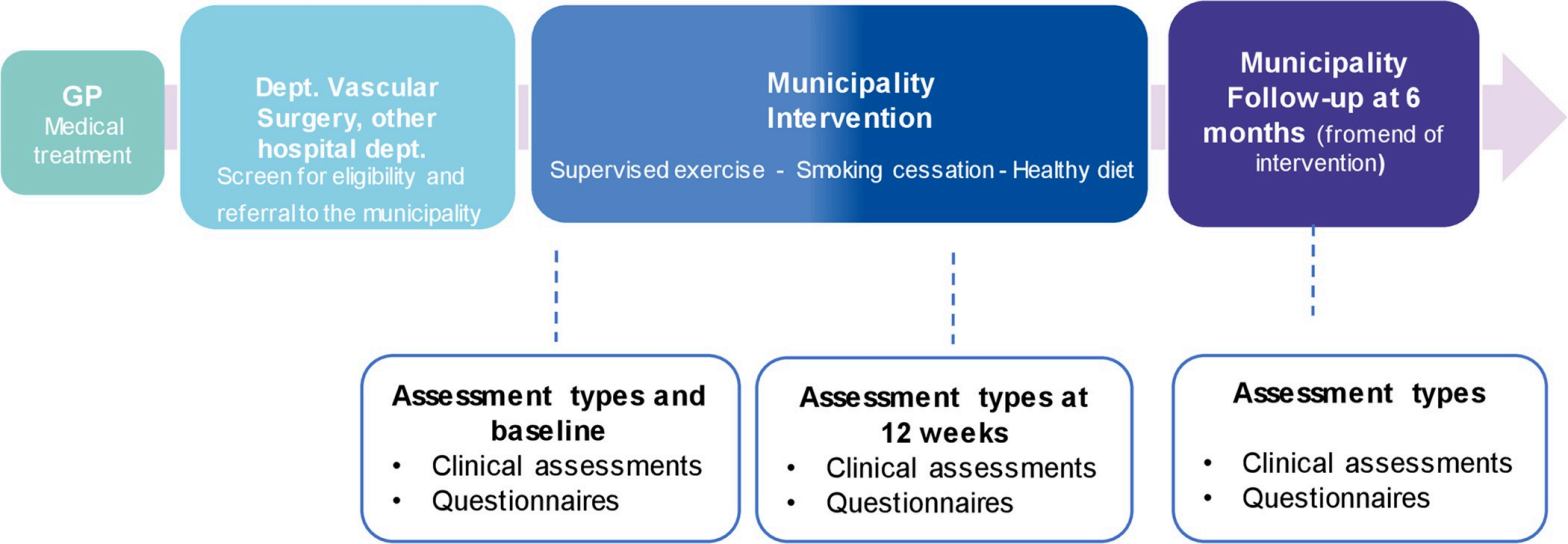
Patient pathways and assessment points.

The flow of patients and data is depicted in Fig 3.

#### Intervention

The SET intervention design is based on the existing evidence on SET for people with IC [2, 6, 7, 16, 25], international guidelines [6], and the experiences from existing interventions in the Southern Region of Denmark [26] and adapted to the context in which the intervention is being implemented in Region Zealand. Each exercise session is supervised in a local healthcare setting as this is found superior to structured home-based exercise therapy and simple walking advice [2]. Further, studies show that exercising in a supervised compared to an unsupervised setting provides further improvement in maximal walking distance and quality of life [7, 8, 27, 28].

The intervention is as follows:

- *Exercise duration and frequency* [2, 6, 25]: 12 weeks supervised exercise training program with three supervised exercise sessions per week. Every session on the treadmill consists of 5 min warm-up, 50 min walk, and 5 min cool down.
- *Modality*: The exercise takes place mainly on the treadmill. The patient must be pushed to walk until they reach 3–4 on the claudication pain scale and then break until the pain are reduced to a minimum (pain score 1) [2, 6].
- *Smoking cessation*: Smoking cessation is essential, and all participants should be screened, and smokers should be referred to smoking cessation [6].
- *Healthy diet*: Guidance to a healthy diet is recommended [6]

Participants are assisted by the physiotherapists in making sustainable plans for future physical activities at the end of the supervised intervention, to maintain the results achieved.

The SET intervention is adapted with guidance from the ADAPT framework [14], with a focus on adaptions to improve intervention-context fit, while maintaining consistency with intervention functions [14].

The intervention is adapted in the following ways: 1) we include participants who are challenged by medical diseases and co-morbidities. Participants not able to walk on the treadmill may attend other types of supervised exercises e.g. cycling. Throughout the intervention the physiotherapists will assess if a participant has raised a suitable capability to walk on the treadmill. 2) The intervention is flexible within the duration, modality and frequency of the SET to meet the organizational setup in each municipality described in more detail below.

The TIDieR checklist has guided the description of the SET intervention [29] as displayed in Table 1.

**Table 1:** The TIDieR checklist. Details of the SET intervention.

Item	Description
1.Brief name	Supervised exercise therapy (SET) and smoking cessation for people with intermittent Claudication.
2. Why	The intervention builds on evidence that is known to have positive effects on health and quality of life [2, 7–9], and this intervention hasn’t been implemented in Region Zealand.
3 What-materials	For the exercise session: a treadmill and a physiotherapist to monitor and guide the participants through the protocol.
4 what-procedures	Before starting the SET, each participant is tested with a 6-min walk and maximal walking distance on a treadmill and is registered in a database. The 12-week program consists of 3 weekly 60-min sessions on a treadmill. Each session consists of 5 min warm up, 50 min training, 5 min cool down. The elevation and pace are adjusted according to the Gardner protocol [30].
5. Who-provided	The supervision and data recording will be delivered by the physiotherapists in each municipality, supervised and trained in delivering the intervention by the project team.
6 How	The exercise will be performed on a treadmill, individually or in smaller groups, each participant on their own treadmill. Supervised by a physiotherapist.
7. Where	In the municipalities, in a local health center in region Zealand, Denmark.
8. When and how much	A total of 36 sessions, consisting of 60 min SET each, are delivered over a 12-week period. Each participant is tested on the treadmill using a protocol (Gardner) to determine speed and elevation. At each session, speed and elevation can be adjusted, and participants rest several times as needed to overcome leg-pain. The Claudication pain scale is used to guide the participants as to when to keep on walking and when to rest.
	The physiotherapists will during the sessions supervise (encourage, monitor and guide) the participants.
9. Tailoring	The program is tailored to each participant using the Gardner protocol as well as the claudication pain scale. The supervision by the physiotherapists is to ensure that each participant is supported and guided to their individual maximum in each session.
10 Modifications	Modifications to the program will be closely monitored and reported.
11. How well (planned)	Physiotherapists delivering the program will receive on-site instruction on how to test and perform the SET. Physiotherapists delivering the program will attend at least one on-site session addressing how to conduct the clinical tests and perform SET as well as on-site supervision. Further in-person or online meetings will be arranged with anyone who finds it necessary.
12 How well (actual)	Data from the SET and from any reported modifications (adaptions) are monitored and will be a part of the results that are disseminated to all stakeholders in the project.

### Supervised exercise therapy

The description of the exercise therapy adheres to the CERT checklist [31].

To set a suitable pace and intensity for each participant and to establish a baseline outcome parameter, the participant performs a treadmill test as described in the Gardner protocol [30]. The test and exercise instructions sheets can be found in the (S1A, S1B Fig).

The SET intervention is inspired by the exercise protocol by Bronas et al. [25]. Each exercise session builds on a 5-minute warm-up, up to 50 minutes of supervised exercise on the treadmill and a 5-minute cool-down. Each session is divided into intervals, each interval conducted until the participant reaches leg pain corresponding to 3–4 on the claudication pain scale (see S2 Fig). In between intervals, participants will take a break until the pain subsides to 1 on the pain scale. The pain scale score is the only assessment tool to monitoring load and stress during the exercise. Progression of the treadmill elevation and pace is adjusted at the following session to ensure the right intensity as described in the exercise instructions sheets (S1B Fig). If participants are not able to accommodate the prescribed walking pace, modifications are possible to suit their individual needs.

SET will, as standard, be performed on a treadmill since the amount of evidence for this exercise modality is strongest. However, alternative exercise modes can be applied when SET on a treadmill is not an option [32]. The SET intervention will be adapted and performed in various ways depending on the organization and facilities of each of the included municipalities. This is allowed to secure local adaptation and implementation of the SET intervention and will be planned in cooperation with the pilot group e.g. the representatives from the municipalities.

Possible adaptions are:

*The frequency* of SET can be twice a week. The participants should be encouraged to walk the third session in their home environment.

*Duration* can be less than 12 weeks, with a minimum of 6 weeks [2, 6].

*Modality* can be altered if the participant is not able to walk on a treadmill due to limited walking abilities etc., other types of supervised exercise activities can be performed e.g. biking [2, 6].

#### Smoking cessation and a healthy diet

Smoking cessation is class-A recommended in the treatment of IC [6] due to substantial evidence showing that continuous smoking exacerbates PAD symptoms and increases the risk of critical ischemia and amputation (25,27). Smoking cessation guidance is therefore important in combination with SET intervention. Smoking cessation programs can be designed differently according to the organization of each municipality, but participants need support to stop smoking [33]. Programs can be individual, group-based, or a combination of the two. They can be with physical attendance, by telephone or online sessions [34]. It is recommended that a professional (e.g. nurse) with an education as a quit smoking counsellor conducts the sessions [35]. Focus on a healthy diet is recommended in the European guidelines [6] as a way to manage risk factors concerning IC. Yet focus on a healthy diet will only be part of the intervention if the municipality can do so.

#### Data management

The quantitative data are collected and managed using REDCap (Research Electronic Data Capture) [36, 37]. REDCap facilitates secure and effective data collection. Data will be collected on all patients with IC referred to SET in the involved municipalities. The REDCap database is administered by ‘The research and implementation unit PROgrez’ at Næstved-Slagelse-Ringsted Hospitals, which leads the implementation process. The qualitative data are collected and stored in a secure drive, managed by the Næstved-Slagelse-Ringsted Hospitals.

#### Data collection

In line with the ADAPT guideline [14], we employ multiple data collection approaches to evaluate the process of the adapted intervention, report outcomes, and assess and compare the intervention’s effects across the different municipalities and with effects demonstrated in clinical trials [2, 7, 16]. The quantitative data will be analysed using various types of regression analysis. However, the specific type of analysis will depend on the exact number of participants and outcomes of interest. A statistical analysis plan will be published before any future analysis are conducted. Data collection will be done at baseline, 12 weeks from baseline and 6 months from end of intervention. Nine months are chosen to give participants an extended period to integrate and show maintenance of the exercise therapy in their everyday lives. According to the Stage of Change model, which describes the process of behaviour change, 6 months from end of intervention is enough for evaluating such change [38].

A range of different collection methods are used and outlined below. They include objectively measured outcomes, patient-reported outcomes, harm re-referrals and dropouts, performance indicators, qualitative evaluation, and organizational data, including economics within each organization. The physiotherapists (assessors) are responsible for informing participants on data use, creating patient files in the database, referrals, reporting physical test data, dropouts, and acceptance of smoking cessation as well as exercise maintenance.

Table 2 is a list of the most essential outcomes, e.g. clinically assessed outcomes and PROMs for HRQoL, well-being, mental health, and healthy lifestyle improvements including instruments and timing of measurement. A full list is available in the (S1 Table) and includes patient’s characteristics and other outcomes. If relevant, additional outcomes can be added during the implementation process [14].

**Table 2:** Outcome measures and patient reported outcomes.

**CLINICALLY ASSESSED OUTCOMES**		
**Outcomes**	**Instrument**	**Timing of measurement**
Maximum walking distance (primary outcome) *	Treadmill test	Baseline, 12 weeks, 6 months
Pain free walking distance*	Treadmill test	Baseline, 12 weeks, 6 months
Physical function	6-minute walking test	Baseline, 12 weeks, 6 months
**PATIENT REPORTED OUTCOMES**		
**Outcomes**	**Instrument**	**Timing of measurement**
Health-related quality of life	VASCUQoL-6	Baseline, 12 weeks, 6 months
Health-related quality of life	EQ-5D-5L	Baseline, 12 weeks, 6 months
Well-being	WHO-5	Baseline, 12 weeks, 6 months
Anxiety & depression	PHQ-4	Baseline, 12 weeks, 6 months
Pain	Purpose-designed items	Baseline, 12 weeks, 6 months
Comorbidities	Purpose-designed items	Baseline, 6 months
Treatment for comorbidities	Purpose-designed items	Baseline, 6 months
Comorbidities’ influence on every-day-life	Purpose-designed items	Baseline, 6 months
Duration and frequency of rehabilitation services	Purpose-designed items	12 weeks
Other rehabilitation services than supervised exercise training	Purpose-designed items	12 weeks
Satisfaction with received rehabilitation	Purpose-designed items	12 weeks
Applicability of received rehabilitation	Purpose-designed items	12 weeks, 6 months
Barriers to participation	Purpose-designed items	12 weeks
Hospitalization or other doctor contact during rehabilitation and in follow-up period	Purpose-designed items	12 weeks, 6 months
Adverse events	Purpose-designed items	12 weeks, 6 months

* Outcome used as a performance indicator

#### Primary and secondary outcomes

The *primary outcome* is the maximum walking distance (MWD), defined as the distance at which the patient cannot continue walking due to pain. The primary outcome is chosen as it represents a central limitation for patients suffering from IC and will further allow us to compare to the existing evidence [2, 6, 7, 26].

*Secondary outcomes* include pain-free walking distance (PWD), defined as the walking time at which the patient first experienced claudication pain and the Vascular Quality of Life Questionnaire (VASCUQoL-6), an instrument to determine HRQoL in patients with PAD. MWD and PWD are measured through a graded treadmill test as described in the Gardner protocol [30]. Both primary and secondary outcomes are measured at baseline, 12 weeks from baseline and 6 months from end of intervention.

#### Other outcomes

Through the REDCap database, Patient Reported Outcome Measures (PROM) questionnaires will be sent to participants’ digital safe mail at baseline, at 12 weeks and 6 months from end of intervention. Questionnaires will be answered online by the participants and responses are stored automatically in the database upon submission.

#### Maintenance and integration into daily life

The achieved results of exercise and lifestyle interventions tend to decrease over time if changes are not successfully integrated into the participant’s everyday life [39, 40]. Physiotherapists will ask the participants (documented in the REDCap database) at the end of the intervention and 6 months from end of intervention, about the integration and maintenance of exercise and will offer motivational advice to support lifestyle changes. Furthermore, municipalities are encouraged to bring in other community stakeholders in this process, such as patient associations, sports associations, and voluntary groups to assist in the transition from supervised exercise to exercising as a part of everyday life.

#### Harms and dropout

Supervised exercise training is considered safe for patients with IC with a low all-cause complication rate [41]. Information on adverse events during the intervention or dropout from SET, and the cause e.g. death, surgery etc. is registered by the physiotherapists in the REDCap database. At 6 months from end of intervention, the participants will receive an electronic questionnaire concerning contact with the hospital or the general practitioner for further registration of harms. Adverse events will be categorized as serious and non-serious according to the Food and Drug Administration definition [42].

#### Performance indicators

The defined performance indicators are inspired by quality indicators from similar databases and interventions, e.g. the Danish Cardiac Rehabilitation Database [43, 44], smoking cessation data [45] and the Danish Population Survey on Health 2021 [46] as well as research studies on IC [2, 7, 26]. All indicators were debated with stakeholders and if necessary, changed before they were finally decided upon. The performance indicators help ensure a steady assessment of the implementation process and secure a continuous evaluation as recommended by the ADAPT guideline [14].

Performance indicators [47] were presented and discussed and 16 performance indicators were agreed upon by stakeholders from the municipalities and the Department of Vascular Surgery at SUH (surgeons, managers of the physiotherapists, health consultants, smoking cessation consultants and clinical physiotherapists). Of the 16, five are chosen as key performance indicators (KPI). All performance indicators can be found in the (S2 Table), while Table 3 displays the five KPIs. We will evaluate KPIs every three months and all performance indicators every six months for a minimum of four years. As the implementation process proceeds and develops, we may adjust the performance indicators accordingly or add other variables in line with ADAPT guidelines to ensure well-informed adaptations of the intervention into practice [14]. Furthermore, the KPI’s are monitored and if below the thresholds they will be discussed with the stakeholders and initiatives can be initiated to raise the KPI’s. KPI’s will be evaluated both across municipalities and for each municipality separately. If it is possible to reach a high enough number of participants in each municipality, effect evaluation will further be done for each municipality.

**Table 3:** Key performance indicators.

Indicator category	Indicator definition	Sector	Type	Standard
**Referral**	1. Proportion of patients with IC consulted at dept. of vascular surgery referred to municipality-based SET training program	Hospital	Process	≥50%
**Enrollment**	2. Proportion of referred patients with IC enrolled in a SET training course and registered in the database with baseline tests.	Municipality	Process	≥90%
**Retention**	3. Proportion of patients with IC with available baseline tests who complete the program with clinical tests at 6-mo. follow-up (from end of intervention)	Municipality	Process	≥60%
**Questionnaire compliance**	4. Proportion of patients with IC with completed baseline questionnaire who complete and return the 6-mo (from end of intervention) follow-up questionnaire	Municipality	Process	≥60%
**Walking distance**	5. Proportion of patients with IC who increase their MWD from baseline until 6-mo. follow-up (from end of intervention) with a minimum of 120%	Municipality	Result	≥50%

SET, Supervised Exercise Training; IC, Intermittent Claudication; MWD, Maximum Walking Distance.

#### Qualitative investigation

Qualitative methods are applied to enhance our understanding of the pitfalls of the implementation process and what works well from the users’ perspectives. We will investigate experiences and acceptability with the intervention among patients and physiotherapists. We will focus on the user’s experiences with experienced barriers and facilitators and investigate how the patients transfer the lifestyle change into their everyday lives, to be able to make improvements in the various components during the first two years.

Two qualitative studies are planned. The scientific philosophy underpinning these studies is hermeneutic as we wish to understand and interpret experiences within both the intervention and the implementation process [48]. As such, the qualitative studies add perspective and nuances to the quantitative data collected.

The first qualitative study is an interview study to gather knowledge of the patients’ experiences with SET. This study will use semi-structured, in-depth, individual face-to-face interviews [49] with patients followed by a thematic analysis [50]. The semi-structured guide is developed with participation of the physiotherapist, and includes the following questions: How does the patients experience the SET? Has it had a meaning in the patients’ everyday lives and how can the intervention be refined? Inclusion criteria are the patients’ completion of the SET, including baseline and 12-week tests. The patients are recruited at the first appointment with the physiotherapist at their local centre and contacted later by a researcher for the interview. The intention is to include a representative sample of participants from each municipality.

The second qualitative study will aim to explore the causes of dropout and factors that can facilitate participants’ enrolment or stay in the SET.

The study will be done through a semi-structured, in-depth, individual interview study with participants and subsequently, a focus group [51] with physiotherapists incorporating these participant perspectives as themes and discussing them in the focus group to provide further insights on circumstances for dropping out, followed by a thematic analysis [50]. To address the anticipated challenges in commuting due to their IC, this study will use individual interviews with participants in their own home or via telephone [49]. Inclusion criteria for participants are the participant’s dropout at any time in the SET and inclusion criteria for physiotherapists are experience with supervising SET. The participants are recruited at the first appointment with the physiotherapist at their local center and contacted later by a researcher for the interview. The physiotherapists are contacted by interviewer during the implementation process. To develop the semi-structured guide, we will work with a panel of patients to provide insights from a user perspective [18].

#### Organizational data

Yearly, we will collect information from each municipality retrospectively. This is to map the different political organizations in each municipality and to investigate the organization of their specific IC intervention and the associated impact, resources and costs. An electronic questionnaire will be developed in the REDCap database, and each rehabilitation manager of the municipality will be the recipient. The focus is on conveying how each municipality conducts SET for participants with IC. This questionnaire has two main aims: 1) evaluate the impact that the organization has on effect, and 2) conduct cost-benefit analyses of the implementation of SET for people with IC. The questionnaire encompasses e.g. the specifics on resources (personal and location) spent in association with the SET intervention and how maintenance is secured post-SET intervention.

#### Ethics

The project has been notified to the National Research Ethics Committee (EMN-2023-02791) and the Data Protection Agency (reg-035-2023). Referral and participation are voluntary and do not require written consent due to the law of data protection §10 [52, 53]. The law states that the data can be used for research without consent. Upon enrolment, participants will be provided with written and oral information about the processing and use of personal data. Before the qualitative interview, participants are informed orally, provided with written information and sign a written consent. The program will adhere to the principles of the Declaration of Helsinki [54].

## Discussion

This protocol paper outlines the comprehensive implementation process of SET and smoking cessation and describes how we plan to go from a strong evidence base of SET for people with IC and translate and adapt this into everyday life clinical settings. Potentially, it will affect the approximately 10,000 people living with IC in the Region of Zealand and the estimated 1500 new cases of IC each year. Likewise, it may guide and inform others in their attempt to implement a similar solution or inspire other implementation processes across various disease groups and in other countries. We are only familiar with one other paper describing an as comprehensive implementation process of SET to patients with IC [7]. The implementation takes place in the Netherlands where the infrastructure is not directly comparable to the infrastructure in the Danish healthcare system. The Dutch SET intervention is driven by insurance companies with the first 20 SET sessions fully or partly paid by the patients themselves [9]. In this current Danish study, the intervention is facilitated from one sector, the Region, and executed in the municipalities, another sector and it is further free of cost to attend SET for each patient. Hence, the implementation context is expected to be different challenged across to two countries.

The implementation process is planned as flexible and iterative with high stakeholder involvement. When allowing flexibility in the implementation, a plan is necessary for monitoring and securing the quality of the intervention [14]. We therefore plan to conduct a process evaluation to examine the success of the implementation strategy itself [55]. In this, we plan to describe the relationship between interventions and context [14, 56] as we are aware that the inclusion of several healthcare sites may lead to different adapted versions of SET. The process evaluation will address the overall implementation by translating scientific results into practice as a whole and stratified for each of the included municipalities [57]. The process evaluation will follow the MRC framework for conducting and reporting process evaluation studies [57]. In the analyses, cross-cutting qualitative and quantitative results on the implementation (adherence, dose, quality of delivery, participant responsiveness and reach) will be synthesized to gain knowledge on how the implementation is delivered and implemented [58]. An expected barrier that may limit our implementation process is the fact that the SET intervention is facilitated from one sector, the Region, and executed in the municipalities, another sector. However, the cost of the intervention lies in the municipalities and not the hospitals. Another limitation that may interfere with the implementation is the use of treadmills. Even though two treadmills per municipality are financed through this project, the treadmills still require a lot of space, and it can take time for the participants to get acquainted with treadmill walking. Further it is not always possible for participants to continue treadmill walking after SET.

### Dissemination

At least one manuscript with the results of the planned implementation process will be submitted to a peer-reviewed journal. Additionally, separate manuscripts will be submitted for publication in peer-reviewed journals covering both the qualitative and quantitative aspects of the implementation.

## Supporting information

S1 Checklist SPIRIT 2013 checklist: Recommended items to address in a clinical trial protocol and related documents*. (DOCX)

S1 Fig a. 6 min-walk test and treadmill test. b. Exercise instruction sheet.
(ZIP)

S2 Fig The claudication pain scale. (DOCX)

S1 Table Outcome measures and patient demographics. (DOCX)

S2 Table Performance Indicators and key performance indicators for evaluation. (DOCX)

S1 File (PDF)
