# Supplementary figures and images for: Supervised exercise-based rehabilitation for people with intermittent claudication–Study protocol for a Danish implementation process (StRiDE)
Source: PLoS One. 2025 Jan 13;20(1):e0315577. doi: 10.1371/journal.pone.0315577 (PMC11729964; doi:10.1371/journal.pone.0315577)

**S1b. Exercise instruction sheet**

Exercise program – treadmill


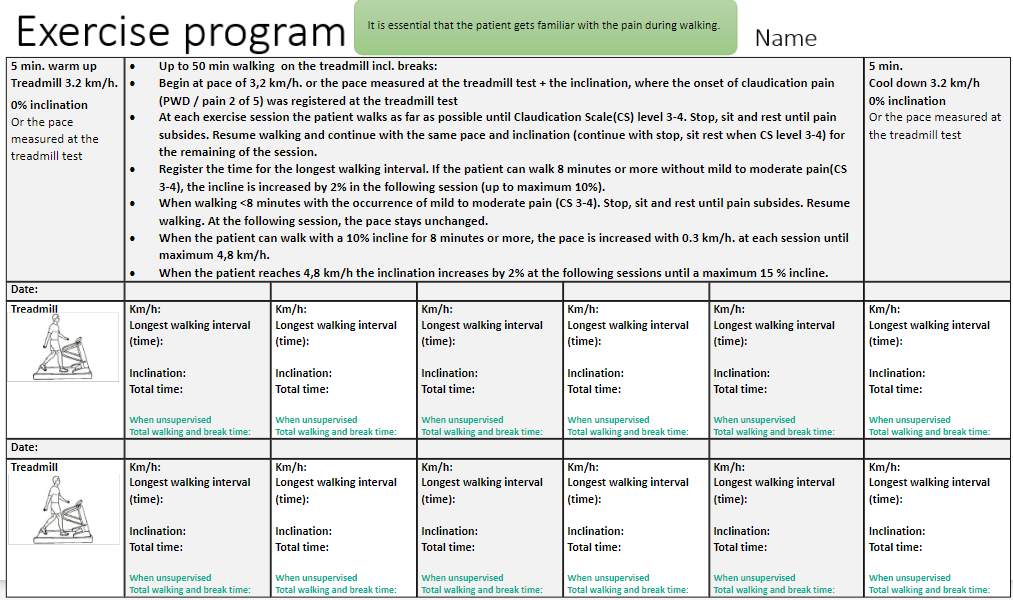

Supplement: S1 Fig — a. 6 min-walk test and treadmill test. b. Exercise instruction sheet. (ZIP) [file pone.0315577.s002.zip › S1b. Exercise instruction sheet.docx]

# **S1a. 6 min-walk test and treadmill test**


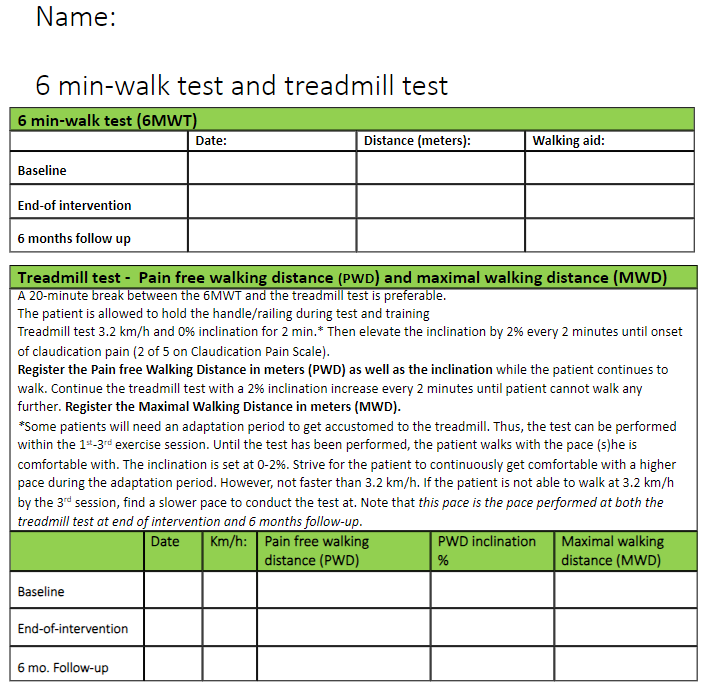

Supplement: S1 Fig — a. 6 min-walk test and treadmill test. b. Exercise instruction sheet. (ZIP) [file pone.0315577.s002.zip › S1a. 6 min-walk test and treadmill test.docx]

# **S2. The Claudication pain scale**


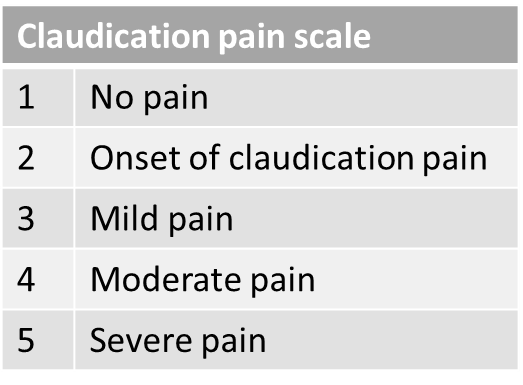

Supplement: S2 Fig — (DOCX) [file pone.0315577.s003.docx]
