# Supplementary material for: Supervised exercise-based rehabilitation for people with intermittent claudication–Study protocol for a Danish implementation process (StRiDE)
Source: PLoS One. 2025 Jan 13;20(1):e0315577. doi: 10.1371/journal.pone.0315577 (PMC11729964; doi:10.1371/journal.pone.0315577)
Supplement: S1 Table — (DOCX) [file pone.0315577.s004.docx]

# **S3. Outcome measures and patient demographics**

A complete list of all outcomes including instruments and timing of measurement

| **Outcome measures and patient demographics** | | |
| --- | --- | --- |
| **CLINICALLY ASSESSED OUTCOMES** | | |
| **Outcomes** | **Instrument** | **Timing of measurement** |
| Maximum walking distance* | Treadmill test | Baseline, 12 weeks, 9 months |
| Pain free walking distance* | Treadmill test | Baseline, 12 weeks, 9 months |
| Physical function | 6-minute walking test | Baseline, 12 weeks, 9 months |
| **PATIENT REPORTED OUTCOMES** | | |
| **Outcomes** | **Instrument** | **Timing of measurement** |
| Health-related quality of life | VASCUQoL-6 | Baseline, 12 weeks, 9 months |
| Health-related quality of life | EQ-5D-5L | Baseline, 12 weeks, 9 months |
| Well-being | WHO-5 | Baseline, 12 weeks, 9 months |
| Anxiety & depression | PHQ-4 | Baseline, 12 weeks, 9 months |
| Pain | Purpose-designed items | Baseline, 12 weeks, 9 months |
| Comorbidities | Purpose-designed items | Baseline, 9 months |
| Treatment for comorbidities | Purpose-designed items | Baseline, 9 months |
| Comorbidities’ influence on every-day-life | Purpose-designed items | Baseline, 9 months |
| Duration and frequency of rehabilitation services | Purpose-designed items | 12 weeks |
| Other rehabilitation services than supervised exercise training | Purpose-designed items | 12 weeks |
| Satisfaction with received rehabilitation | Purpose-designed items | 12 weeks |
| Applicability of received rehabilitation | Purpose-designed items | 12 weeks, 9 months |
| Barriers to participation | Purpose-designed items | 12 weeks |
| Hospitalization or other doctor contact during rehabilitation and in follow-up period | Purpose-designed items | 12 weeks, 9 months |
| Adverse events | Purpose-designed items | 12 weeks, 9 months |
| **PATIENT CHARACTERISTICS AND OTHER OUTCOMES** | | |
| **Outcomes** | **Instrument** | **Timing of measurement** |
| Recruitment* | Number of patients with IC, referred and included | Baseline |
| Completion of SET* | Number of participants who complete rehabilitation | 12 weeks, 9 months |
| Response rate on patient-reported outcomes* | Number of participants returning patient reported outcomes | Baseline, 12 weeks, 9 months |
| Age | Registered by assessor | Baseline |
| Sex | Registered by assessor | Baseline |
| Height | Patient-reported | Baseline |
| Weight | Patient-reported | Baseline, 12 weeks, 9 months |
| Danish language abilities | Registered by assessor | Baseline |
| Citizenship | Patient-reported | Baseline |
| Place of citizenship (municipality) | Patient-reported | Baseline |
| Distance to rehabilitation facility | Patient-reported | Baseline |
| Cohabitation | Patient-reported | Baseline |
| Education | Patient-reported | Baseline |
| Employment status | Patient-reported | Baseline, 9 months |
| Access to digital post | Registered by assessor | Baseline |
| Referral, date | Registered by assessor | Baseline |
| Diagnose for which patient is referred | Registered by assessor | Baseline |
| Referral, origin | Registered by assessor | Baseline |
| Referral, type | Registered by assessor | Baseline |
| Eligibility – SET is relevant and initiated | Registered by assessor | Baseline |
| Smoking | Registered by assessor  Patient-reported | Baseline, 9 months  Baseline, 12 weeks, 9 months |
| Smoking cessation acceptance | Registered by assessor  Patient-reported | Baseline, 12 weeks, 9 months |
| Alcohol consumption | Patient-reported | Baseline, 9 months |
| Exercise and physical activity habits | Patient-reported | Baseline, 12 weeks, 9 months |
| Treatment for IC | Patient-reported | Baseline, 12 weeks, 9 months |
| Plan for exercise maintenance after end-of-intervention | Registered by assessor | 12 weeks |
| Engagement in exercise after end-of-intervention | Registered by assessor | 9 months |
| Drop-out, reasons for drop-out and actions taken | Registered by assessor | At any point in time |
| Repetitive referrals received in municipality** | Registered by assessor | At any point in time |

* Outcome used as a performance indicator.
** Patients are identified in the database as unique cases, thus can only be recorded once. However repetitive referrals will be registered.
