# Supplementary material for: Supervised exercise-based rehabilitation for people with intermittent claudication–Study protocol for a Danish implementation process (StRiDE)
Source: PLoS One. 2025 Jan 13;20(1):e0315577. doi: 10.1371/journal.pone.0315577 (PMC11729964; doi:10.1371/journal.pone.0315577)
Supplement: S2 Table — (DOCX) [file pone.0315577.s005.docx]

# **S4. Performance Indicators and key performance indicators for evaluation**

| **Performance and Key Performance Indicators for the evaluation of the implementation of supervised exercise therapy to patients with intermittent claudication in Region Zealand, Denmark.** | | | | | |
| --- | --- | --- | --- | --- | --- |
| **Indicator category** | **Indicator definition** | **Sector** | **Type** | **Standard** | **How and from where is data extracted** |
| Referral | 1. Proportion of patients with IC consulted at dept. of vascular surgery referred to municipality-based SET training program * | Hospital | Process | ≥ 50% | Data from hospital patient journal system (SP). Diagnose code ***DZ508*** (number of referrals to rehabilitation (GOP)), and ***DI739A*** (all patients with IC in dept. of vascular surgery). |
| Enrolment | 1. Proportion of referred patients enrolled in a SET training course and registered in the database with baseline tests * | Municipality | Process | ≥90% | Data from the municipality patient journal system on no. of patients with IC referred, *and*  Data from the IC database on no. of patients who are enrolled and registered |
| Completion of SET | 1. The proportion of patients with available baseline tests who complete the program with clinical tests at 12-week follow-up. | Municipality | Process | ≥70% | IC database |
|  | 1. Proportion of patients with available baseline tests who complete the program with clinical tests at 6-mo. follow-up * | Municipality | Process | ≥60% | IC database |
| Questionnaire compliance | 1. Proportion of patients who complete and return the baseline questionnaires | Municipality | Process | ≥70% | IC database |
|  | 1. Proportion of patients with completed baseline questionnaire who complete and return the 12-weeks follow-up questionnaire | Municipality | Process | ≥70% | IC database |
|  | 1. Proportion of patients with completed baseline questionnaire who complete and return the 6-mo. follow-up questionnaire * | Municipality | Process | ≥60% | IC database |
| Attendance | 1. Proportion of patients who attend ≥80% of scheduled SET sessions. | Municipality | Process | ≥80% | IC database |
| Walking distance | 1. Proportion of patients who increase their PWD from baseline until 12-weeks follow-up with a minimum of 116% | Municipality | Result | ≥70% | IC database |
|  | 1. Proportion of patients who increase their PWD from baseline until 6 months follow-up with a minimum of 116% | Municipality | Result | ≥50% | IC database |
|  | 1. Proportion of patients who increase their MWD from baseline until 12-weeks follow-up with a minimum of 120% | Municipality | Result | ≥70% | IC database |
|  | 1. Proportion of patients who increase their MWD from baseline until 6 months follow-up with a minimum of 120%* | Municipality | Result | ≥50% | IC database |
| Smoking cessation | 1. Proportion of smokers offered smoking cessation support minimum twice during the 12-week course | Municipality | Process | ≥90% | IC database |
|  | 1. Proportion of smokers who are offered smoking cessation, accept support within the 12-week course | Municipality | Process | ≥50% | IC database |
|  | 1. Proportion of smokers that have quit smoking at 12-week follow-up | Municipality | Result | ≥40% | IC database |
|  | 1. Proportion of smokers that have quit smoking at 6 months follow-up | Municipality | Result | ≥30% | IC database |
| *Key Performance Indicator.  SET: Supervised Exercise Training; IC: Intermittent Claudication; PWD: Pain Free Walking Distance; MWD: Maximum Walking Distance | | | | | |
