# Supplementary material for: Supervised exercise-based rehabilitation for people with intermittent claudication–Study protocol for a Danish implementation process (StRiDE)
Source: PLoS One. 2025 Jan 13;20(1):e0315577. doi: 10.1371/journal.pone.0315577 (PMC11729964; doi:10.1371/journal.pone.0315577)
Supplement: S1 File — (PDF) [file pone.0315577.s006.pdf]

Project description: Implementation of municipality based supervised exercise for citizens with intermittent claudication

Objective

To implement evidence-based supervised exercise training in a municipality-based health care setting to all citizens with intermittent claudication in the Region of Zealand, Denmark.

Background

European guidelines recommend that all patients with intermittent claudication should undergo a comprehensive intervention combining supervised exercise training (SET) and smoking cessation as part of first line treatment (1). The recommendation builds on solid evidence showing that supervised exercise training (SET) and smoking cessation can increase walking distance and reduce pain in people with intermittent claudication (IC) (2,3). Further, SET is found to be an alternative to surgery (2,4). The CLEVER protocol (3,4) found, that supervised exercise training in combination with optimal medical treatment was significantly superior compared to both medical treatment alone and medical treatment combined with stent revascularization when measured on walking distance after 6 months.

In Denmark, there are no national guidelines on the treatment of IC although the general advice is for patients to walk more and stop smoking. Consequently, Danish IC patients rarely receive the treatment recommended in international guidelines (1). Only a few municipalities across Denmark provide systematic SET targeting IC patients, but in a majority of Denmark IC patients are left with no professional supervision.

It is essential that IC patients' exercise in a supervised setting, as it is crucial to exercise through pain – something that is psychologically challenging without guidance (2,5,6).

Surveys from 2018 have shown that vascular surgeons in Denmark would refer their patients to SET if it was an option (7). Since 2019 the Region of Southern Denmark have implemented SET to their IC patients, based on the CLEVER protocol (8). The SET intervention has a duration of 12 weeks. Patients are diagnosed and referred to municipality setting for SET by a vascular surgeons in alignment with international guidelines (1). More recent experiences, specifically from Fredericia Municipality, have showed good results and a high satisfaction level among the enrolled patients.

Despite the evidence and clinical experiences, the delivery of intervention is still limited. In 2022, however, the Regional Council of Zealand allocated funds to implement SET as part of routine practice to citizens with intermittent claudication in all of its 17 municipalities.

The aim with the implementation is that all citizens with intermittent claudication in Region Zealand are referred to a comprehensive life-style course including supervised exercise training close to their residence and that all 17 municipalities have a course to match those referrals.

Methods

Study design

The Medical Research Council's framework for developing and evaluating complex intervention (9) forms the theoretical foundation for the implementation project. While the body of evidence is already heavily supporting SET and smoking cessation (2) a successful implementation across various settings and services still requires a feasibility period before then moving to larger scale implementation of the intervention.

Hence, the implementation process has different stages starting with a pilot phase in four municipalities prior to enrolling the intervention in all 17 municipalities. This will allow, in a prospective cohort design, for

an ongoing assessment of feasibility, adjustments of the intervention to fit the context, as well as ongoing evaluation on the effect and impact of the intervention with the aim to find significant effect in patients' physical performance, on one hand, and an intervention that is feasible in each municipality's unique context.

Target group

People with intermittent claudication who live in the Region of Zealand and who are believed to benefit from an exercise intervention.

The department of vascular surgery and general practitioners will be responsible for screening for eligibility among IC patients and referring relevant patients to the municipality, who are responsible for offering the patient a SET program including help to smoking cessation.

Setting

The SET intervention will take place in the rehabilitation centers in the municipality where the IC patient lives. The implementation will fold out in a close collaboration between the department of vascular surgery at University Hospital Zealand and the rehabilitation centers. There are 17 municipalities in region Zealand, some of which have more than one rehabilitation center.

The SET intervention is based on the CLEVER protocol (3,4), international guidelines (1) and the experiences of the Southern Region of Denmark and their implementation of SET to IC patients as described in the background section.

In the current protocol, an optimal supervised exercise training means:

- **Duration and frequency**: 12 weeks program with 3 sessions per week.
- **Modality**: The exercise take place mainly on treadmill as it is essential that the patient is pushed to walk beyond pain threshold. One of the three weekly sessions is outdoor to increase transferability.
- **Smoking cessation and lifestyle:** smoking cessation and supervision and guidance in healthy lifestyle is an integrated part of the program. This is because smoking cessation is the most important factor for this patient group and continuous smoking will exacerbate symptoms and increase the risk of amputation.

Each SET session builds on 5-minute warm-up, 50 minute SET, 5-minute cool-down and patients can follow either pace 3.2 km/h or 2.0 km/h depending on the results of their baseline 6-minute walking test (6mwt). SET is performed in intervals of 8 minutes with the aim to always reach leg pain corresponding to 2-3 on the claudication pain scale. In between intervals, patients will take a break until pain disappears.

Pace is set as follows:

- Protocol 3.2 is used if patient walks ≥ 300 meter on 6MWT
- Protocol 2.0 is used if patient walks < 300 meter on 6MWT

The treadmill elevation is then set to ensure the right intensity. Elevation can be increased by 2% increments (until maximum 10%). If reaching a 10% elevation, pace is adjusted with 0.2 km/h (until maximum 4.8 and 3.2 km/h for each protocol respectively). If reaching maximum pace, elevation can be adjusted again, now in 1-2% increments until maximum 15%.

To accommodate the complexity of reality and each municipality's resources, physical framework and in all matters unique setting the rehabilitation centers are not required to strictly implement the protocol as

outlined above. This is why data collection on the organization of each municipality's IC program becomes important to evaluate which factors restricts and facilitates improved health data.

Study outcomes

To evaluate the effect of the intervention the primary end-point is maximal walking distance (MWD) measured on a graded treadmill test by the Gardner protocol (10) 12 weeks after baseline. Secondary outcomes are pain-free walking distance (PWD), walking pace, quality of life (QoL) and healthy lifestyle improvements (smoking cessation, weight loss).

To evaluate the success of the implementation we firstly look at referral statistics, e.g. are patients referred to SET in their municipality, do referred patients enroll in a SET program and do enrolled patients adhere to the prescribed exercise regimen. Secondly, we will attend to the effect of the intervention as per the outcomes outlined above.

Data is collected at baseline, 12 weeks (end-of-SET) and 9 months (6 months after end-of-SET).

On a yearly basis, there will be collected information from each municipality retrospectively on the organization of their specific IC intervention and the resources and costs it has for them. This is to 1) evaluate the significance of organization on effect, and 2) conduct cost-benefit analyses of the implementation of SET.

Lastly, there will be performed qualitative interviews with patients, physiotherapists and surgeons to gather in-depth knowledge on pitfalls and potential for improvements of the intervention and the implementation.

Table 1 shows the categories of data being collected and when, as well as who is responsible for reporting the data to the database.

Table 1

Type of data	Baseline	12 weeks	9 months	Reported by
Identification (name, CPR)	X			Therapist
Weight and height	X	X	X	Therapist
Physical function (6 minute walking test + graded treadmill)	X	X	X	Therapist
Demographic data (age, sex, marital, work, education)	X			Patient
Health and treatment (anxiety, depression, surgery, medical treatment, comorbidities)	X	X	X	Patient
Lifestyle (exercise, smoking, alcohol)	X			Patient
QoL, (VASCUQoL-6, WHO-5, EQ-5D)	X	X	X	Patient
Motivation (PAM)	X			Patient
Adherence to intervention		X		Patient
Organizational and financial data				Municipality; therapist/management
Qualitative interviews				Patients Physio therapists Surgeons

Data collection

Data will be collected and managed using the REDCap electronic data capture tools (11,12) – approved by the Region of Zealand. REDCap facilitates secure and effective data collection. Data will be collected on all IC patients that enroll in a SET program in their municipality and who agree to have their data collected and stored.

Data is collected with the purpose of 1) evaluate the implementation, 2) evaluate the effect of the intervention and 3) create a database for future IC research to improve treatment to benefit patients and society.

Data will primarily exist of patient reported outcome measures (PROMs). The physical assessment is performed by the physical therapists in the municipality who will also be responsible for collecting declaration of consent before baseline assessment, create the patient file in the database and report physical test data and drop-outs.

The database is developed and owned by the research department PROgrez.

The figure 1 below illustrates the data collection process.

Figure 1

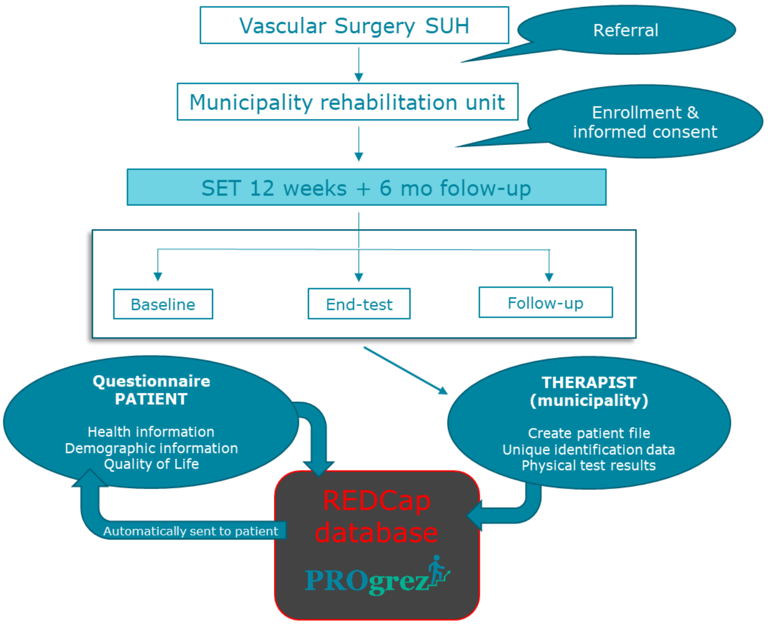

Ethical considerations

Implementing SET to patients with IC will improve standard care. International guidelines recommend SET as a first choice treatment in combination with medication, and evidence is profound that SET is the optimal treatment for patients with IC (1,2). Despite a large body of research that have established this, Denmark has no national guidelines on this today. Surveys have shown that surgeons are willing to refer

relevant patients to evidence based SET programs if they exist (7). Vascular surgeons are in high demand and waiting lists necessitates that surgeons prioritize patients with critical ischemia, often leaving IC patients on their own.

The project will conform to the principles of the Declaration of Helsinki. Patients who are referred to and enroll in a municipality SET program will not be restricted from participating in any activities or treatments outside the intervention. SET is considered safe for patients with IC with a low all-cause complication rate (13). Participants will likely benefit from SET both in the short and the long term by preventing and/or postponing the need for vascular surgery.

The implementation will ensure voluntary participation and the right to the best treatment available. Participants will be provided written and oral information upon enrolment and data will only be collected and stored upon informed consent. The program will be notified to the National Research Ethics Committee and the Danish Data Protection Agency.

Information concerning participants will be protected according to the "Act on Processing of Personal Data" (i.e. Persondataloven) as well to the "Act on Health". All staff associated with the implementation, who are also subject to professional secrecy, will treat data confidentially.

Organization and time plan

By 2023 the 17 municipalities in Region Zealand was divided in four clusters each associated with a local hospital. To accommodate the newly established cluster collaboration a municipality from each cluster were appointed pilot municipalities. The first municipalities (Næstved, Køge, Vordingborg) is expected to enroll their first IC patients for SET in May 2023 with Kalundborg following in September.

The program will be enrolled to the remaining 13 municipalities during 2024.

PROgrez, a research unit in the department of physio- and occupational therapy and the hospital of Slagelse, is responsible for project management and the evaluation of the implementation, including data collection and storage.

Dissemination

An implementation protocol and all research findings related to the implementation will be published in relevant peer-reviewed journals.

Moreover, there will be created reports in aggregated form on municipality level for the municipalities to use in their documentation and quality improvement.

Perspective

The central premise to a supervised exercise regime is that for patients to push beyond pain threshold they need supervision. To most people it is contra-intuitive to induce pain on themselves and thus IC patients typically end up in a sedentary life-style that only worsen their peripheral artery disease and their general health. For this vulnerable patient group with high prevalence of lifestyle-related comorbidities and progression to critical ischemia the potential benefit of enrollment in a SET program will be both short- and long term.

Referencer

1. Aboyans V, Ricco JB, Bartelink MLEL, Björck M, Brodmann M, Cohnert T, m.fl. 2017 ESC Guidelines on the Diagnosis and Treatment of Peripheral Arterial Diseases, in collaboration with the European Society for Vascular Surgery (ESVS). European Heart Journal. 1. marts 2018;39(9):763–816.

2. Hageman D, Fokkenrood HJP, Gommans LNM, van den Houten MML, Teijink JAW. Supervised exercise therapy versus home-based exercise therapy versus walking advice for intermittent claudication. Cochrane Database of Systematic Reviews. 6. april 2018;2018(4).

3. Murphy TP, Cutlip DE, Regensteiner JG, Mohler ER, Cohen DJ, Reynolds MR, m.fl. Supervised exercise versus primary stenting for claudication resulting from aortoiliac peripheral artery disease: Six-month outcomes from the claudication: Exercise versus endoluminal revascularization (CLEVER) study. Circulation. 3. januar 2012;125(1):130–9.

4. Murphy TP, Hirsch AT, Ricotta JJ, Cutlip DE, Mohler E, Regensteiner JG, m.fl. The CLEVER (Claudication: Exercise Vs. Endoluminal Revascularization) Study: Rationale and Methods.

5. Galea MN, Bray SR, Ginis KAM. Barriers and facilitators for walking in individuals with intermittent claudication. J Aging Phys Act. januar 2008;16(1):69–83; quiz 84.

6. Galea Holmes MN, Weinman JA, Bearne LM. "You can't walk with cramp!" A qualitative exploration of individuals' beliefs and experiences of walking as treatment for intermittent claudication. Journal of Health Psychology. 1. februar 2017;22(2):255–65.

7. Jensen JD, Tang LH, Zwisler ADO, Houlind KC, Skou ST. National survey of current practice and opinions on rehabilitation for intermittent claudication in the Danish Public Healthcare System. Scandinavian Cardiovascular Journal. 2. november 2019;53(6):361–72.

8. Jacobsen A, Houlind KC, Rai A. Life-style counseling program and supervised exercise improves walking distance and quality of life in patients with intermittent claudication. Physiotherapy Theory and Practice. 18. november 2022;38(13):2629–39.

9. Skivington K, Matthews L, Simpson SA, Craig P, Baird J, Blazeby JM, m.fl. A new framework for developing and evaluating complex interventions: Update of Medical Research Council guidance. The BMJ. 30. september 2021;374.

10. Gardner AW, Skinner JS, Cantwell BW, Smith LK. Progressive vs single-stage treadmill tests for evaluation of claudication. Med Sci Sports Exerc. april 1991;23(4):402–8.

11. Harris PA, Taylor R, Thielke R, Payne J, Gonzalez N, Conde JG. Research electronic data capture (REDCap)-A metadata-driven methodology and workflow process for providing translational research informatics support. Journal of Biomedical Informatics. 2009;42(2):377–81.

12. Harris PA, Taylor R, Minor BL, Elliott V, Fernandez M, O'Neal L, m.fl. The REDCap consortium: Building an international community of software platform partners. J Biomed Inform. juli 2019;95:103208.

13. Gommans LNM, Fokkenrood HJP, van Dalen HCW, Scheltinga MRM, Teijink JAW, Peters RJG. Safety of supervised exercise therapy in patients with intermittent claudication. J Vasc Surg. februar 2015;61(2):512-518.e2.
